# Assessing Needs, Well-being, and Telehealth Access of Older Adults with Limited Resources during COVID-19 Pandemic

**DOI:** 10.1093/geroni/igab046.2725

**Published:** 2021-12-17

**Authors:** Sashah Damier, Assma Twahir, Sweta Tewary, Naushira Pandya, Nicole Cook

**Affiliations:** 1 NSU, Davie,FL, Florida, United States; 2 NSU, Davie, Florida, United States; 3 Nova Southeastern University, Fort Lauderdale, Florida, United States

## Abstract

The COVID-19 pandemic created new barriers and challenges to accessing primary care services, particularly among older adults who already faced barriers related to access to care, including transportation, health literacy, and self-management support. Nova Southeastern University South Florida Geriatric Workforce Enhancement Program (NSU SFGWEP) partnered with primary care clinics to conduct wellness calls to older adult patients identified through clinic EHR. The wellness calls’ objectives were to 1) discuss COVID-19 protective measures; 2) assess wellness needs and access to care barriers; and 3) screen for telehealth support. From September 2020 to February 2021, the team (including medical students, public health students, and SFGWEP staff) contacted 200 patients via telephone and conducted a comprehensive wellness survey developed by the study team, informed by validated surveys. Among the 200 patients called, 60% (n=34) were very concerned about the Covid-19 pandemic, 33% (n=34) reported often feeling isolated from others, and 20% (n=34) expressed difficulty getting medical care. A smaller subset of patients reported concern about limited COVID-19 testing (n=1), lack of knowledge about seeing their provider via telehealth (n=7), lack of face masks (n=1), and challenges with obtaining medication refills (n=1). Following wellness calls, the NSU SFGWEP team provided education, referred to clinical resources, and, for low-income patients with access to care challenges, provided Samsung Tablets (n=50) with peer training to enable telehealth.

